# Blockade of histamine receptor H1 augments immune checkpoint therapy by enhancing MHC-I expression in pancreatic cancer cells

**DOI:** 10.1186/s13046-024-03060-5

**Published:** 2024-05-08

**Authors:** PingShan Zhong, Kohei Nakata, Koki Oyama, Nobuhiro Higashijima, Akiko Sagara, Satomi Date, HaiZhen Luo, Masataka Hayashi, Akihiro Kubo, ChenYi Wu, Shan He, Takeo Yamamoto, Kazuhiro Koikawa, Chika Iwamoto, Toshiya Abe, Naoki Ikenaga, Kenoki Ohuchida, Takashi Morisaki, Yoshinao Oda, Keiji Kuba, Masafumi Nakamura

**Affiliations:** 1 Department of Surgery and Oncology, Graduate School of Medical Sciences, Kyushu University, Fukuoka, 812-8582 Japan; 2 Department of Diagnostics and Therapeutics Endoscopy, Kyushu University Hospital, Fukuoka, 812-8582 Japan; 3 Department of Overseas Exchange Center, Kyushu University Hospital, Fukuoka, 812-8582 Japan; 4 Department of Anatomic Pathology, Pathological Sciences, Graduate School of Medical Sciences, Kyushu University, Fukuoka, 812-8582 Japan; 5 Department of Cancer Immunotherapy, Fukuoka General Cancer Clinic, Fukuoka, 812-0018 Japan; 6 Department of Pharmacology, Graduate School of Medical Sciences, Kyushu University, Fukuoka, 812-8582 Japan

**Keywords:** Pancreatic cancer cell, Histamine receptor H1, Major histocompatibility complex class I

## Abstract

**Background:**

Although immune checkpoint blockade (ICB) therapy has proven to be extremely effective at managing certain cancers, its efficacy in treating pancreatic ductal adenocarcinoma (PDAC) has been limited. Therefore, enhancing the effect of ICB could improve the prognosis of PDAC. In this study, we focused on the histamine receptor H1 (HRH1) and investigated its impact on ICB therapy for PDAC.

**Methods:**

We assessed HRH1 expression in pancreatic cancer cell (PCC) specimens from PDAC patients through public data analysis and immunohistochemical (IHC) staining. The impact of HRH1 in PCCs was evaluated using HRH1 antagonists and small hairpin RNA (shRNA). Techniques including Western blot, flow cytometry, quantitative reverse transcription polymerase chain reaction (RT-PCR), and microarray analyses were performed to identify the relationships between HRH1 and major histocompatibility complex class I (MHC-I) expression in cancer cells. We combined HRH1 antagonism or knockdown with anti-programmed death receptor 1 (αPD-1) therapy in orthotopic models, employing IHC, immunofluorescence, and hematoxylin and eosin staining for assessment.

**Results:**

HRH1 expression in cancer cells was negatively correlated with HLA-ABC expression, CD8^+^ T cells, and cytotoxic CD8^+^ T cells. Our findings indicate that HRH1 blockade upregulates MHC-I expression in PCCs via cholesterol biosynthesis signaling. In the orthotopic model, the combined inhibition of HRH1 and αPD-1 blockade enhanced cytotoxic CD8^+^ T cell penetration and efficacy, overcoming resistance to ICB therapy.

**Conclusions:**

HRH1 plays an immunosuppressive role in cancer cells. Consequently, HRH1 intervention may be a promising method to amplify the responsiveness of PDAC to immunotherapy.

**Supplementary Information:**

The online version contains supplementary material available at 10.1186/s13046-024-03060-5.

## Background

Pancreatic ductal adenocarcinoma (PDAC) ranks as the third leading cause of cancer-related deaths, with significantly worse survival rates than other solid malignancies [1, 2] due to late-stage diagnosis and multidrug resistance [3]. Recent advancements in immune checkpoint blockade (ICB) have markedly improved the treatment of cancers with poor prognoses, such as lung cancer and melanoma. PDAC is generally regarded as an “immunologically cold” tumor due to the immunosuppressive tumor microenvironment (TME), which can limit the therapeutic effect of ICB [4]. However, patients with microsatellite instability (MSI)-high PDAC respond exceptionally well to ICB [5], and increased tumor-infiltrating CD8^+^ T cells correlate with improved PDAC survival [6]. This suggests that ICB could be effective under specific conditions.

CD8^+^ T cells recognize tumor antigens presented on major histocompatibility complex (MHC) class I, mediating ICB responses [7]. There is growing interest in understanding the role of MHC-I in cancer cells, particularly due to its loss or decrease in expression in various cancer types, including PDAC [8–10]. This reduction in MHC-I may contribute to cancer cells evading immune surveillance. Increased surface MHC-I expression has demonstrated improved immunotherapy outcomes in melanoma mouse models [11]. Therefore, enhancing MHC-I expression in cancer cells may be a promising treatment method for enhancing immune identification and targeting PDAC [9, 12, 13].

Histamine is a physiological molecule that classically mediates inflammatory responses by binding to histamine receptors such as histamine receptors H1, 2, 3, and 4 [14]. Recently, interest in the relationship between antihistamines and immune cells has increased, and the clinical use of antihistamines has been reported to be associated with improved survival in patients undergoing immunotherapy [15, 16]. Histamine binding of histamine receptor H1 (HRH1) to macrophages induces an immunosuppressive phenotype. Treatment with H1-antihistamine enhances the immunotherapy response, inhibiting cancer development and improving survival in mouse breast cancer and melanoma models [17]. These findings have sparked increased interest in HRH1 antagonists as potential candidates for combination immunotherapies against tumors. However, the understanding of the relationship between HRH1 and the immune system in PDAC is currently limited.

Here, we hypothesized that HRH1 is related to tumor immunity in PDAC, and combining its antagonist with ICB may enhance the effectiveness of pancreatic cancer treatment. Additionally, we aimed to identify a novel immune activation mechanism for HRH1 inhibition in PDAC.

## Methods

### Public data and analysis

The Cancer Genome Atlas (TCGA) and other publicly available datasets, such as the Gene Expression Omnibus (GEO) and Genotype-Tissue Expression (GTEx), were used to obtain HRH1 mRNA levels in PDAC, normal pancreatic tissues, and adjacent non-tumor tissues. The mRNA expression levels of HRH1 were obtained using the R2 Genomics Analysis and Visualization Platform (R2) (http://r2.amc.nl). The total survival rate associated with HRH1 expression was determined via R2. Assessment of HRH1 mRNA levels and analysis of T cell exhaustion scores were conducted using GEPIA 2.0. Additionally, single-cell RNA sequencing data (Genome Sequence Archive [GSA]: CRA001160) was used to investigate the correlation between HRH1 expression and immune cells.

### Tissue samples from patients with PDAC, immunohistochemical (IHC) staining, and assessment

Human PDAC tissue samples utilized in this research were acquired from a cohort of 3 patients who underwent surgical resections for pancreatic cancer at Kyushu University Hospital in Fukuoka, Japan. The tissues were sectioned into 4 μm sections and subsequently incubated overnight at 4°C with the following antibodies: rabbit anti-HRH1 antibody (1:100, #13413–1-AP; Proteintech, Chicago, USA), mouse anti-CD8 antibody (#413201; Nichirei Biosciences, Tokyo, Japan), mouse anti-HLA-ABC antibody (1:500, #ab70328; Abcam, Cambridge, UK), and mouse anti-Granzyme B antibody (1:100, #SC-8022; Santa Cruz Biotechnology, California, USA). Immunohistochemical staining was performed using specific antibodies for HRH1, CD8, HLA-ABC, and Granzyme B and stained accordingly with EnVision System-HRP-l Labeled Polymer Anti-Rabbit antibody (#K4003; Dako, California, USA) or EnVision System-HRP-l Labeled Polymer Anti-Mouse antibody (#K4001; Dako, California, USA). The procedures were conducted using consecutive slices. The BZ-X Analyzer (Keyence 700 or 800) was used for image processing and quantification.

Staining intensity was assessed at 200 × magnification to compare the presence of HRH1-positive cancer cells with that of normal ducts in the corresponding sections. Staining intensity was categorized into four distinct groups: 0 (absent), 1 (low), 2 (moderate), and 3 (high). HRH1 or HLA-ABC intensity ≥ 2 was considered a high expression, while < 2 was assigned a low expression. In addition, we evaluated the quantity of CD8-positive or Granzyme B-positive cells in a minimum of three distinct tumor locations per slice using a magnification of 200 × . CD8 expression was assessed using a grading system and categorized as either high or low. CD8 expression was considered high if the count of positive cells per high-magnification field (200 ×) exceeded 100. Granzyme B levels were assessed based on their expression levels in pancreatic cancer tissues. If Granzyme B exhibited low expression or was undetected, it was labeled as negative; otherwise, it was considered positive.

### Cells and reagents

Human cancer-associated fibroblasts (CAF1, CAF2, and CAF3) were derived from human pancreatic cancer tissues. Mouse cancer-associated fibroblast CAF (mouse CAF1) and seven cancer cell lines (KPC-1 to KPC-7) were generated from the primary pancreatic tumors of KPC mice. An outgrowth technique [18, 19] was used for this purpose. The human cancer cell lines AsPC-1, BxPC-3, SW 1990, U2OS, MCF-7 (American type culture collection, Manassas, Virginia, USA), SUIT-2, MIA PaCa-2, KP-2 (Japan Health Science Research Resources Bank, Osaka, Japan), and PANC-1 (Riken BioResource Center, Ibaraki, Japan) were procured and cultured in Dulbecco's modified Eagle's medium (DMEM) supplemented with 10% fetal bovine serum (FBS) at 37°C with 10% CO_2_, as previously described [20]. THP-1 (Japan Health Science Research Resources Bank, Osaka, Japan) was purchased and cultured in Roswell Park Memorial Institute (RPMI) 1640 medium supplemented with 10% FBS at 37 °C with 5% CO_2_. Before performing any animal experiments, all cell lines were thoroughly checked for mycoplasma contamination and found to be negative. The reagents used in this study were azelastine hydrochloride (Az) (#79307–93-0; Tokyo Chemical Industry, Tokyo, Japan), promethazine hydrochloride (Pt) (#58–33-3; Tokyo Chemical Industry, Tokyo, Japan), desloratadine (Da) (#100643–71-8; Tokyo Chemical Industry, Tokyo, Japan), simvastatin (sim) (#S6196; Sigma-Aldrich), and phorbol 12-myristate 13-acetate (PMA) (#16561–29-8; Abcam, Cambridge, UK). These reagents were dissolved in sterile water (H_2_O) or dimethyl sulfoxide (DMSO) to a concentration of 10 mM. We also used histamine dihydrochloride (#H7250; Sigma-Aldrich, Burlington, Massachusetts, USA), recombinant human IL-4 (#204-IL; Bio-Techne, Minneapolis, Minnesota, USA), recombinant mouse IL-4 (#214–14; Pepro Tech, Cranbury, NJ, USA), recombinant mouse granulocyte–macrophage colony-stimulating factor (GM-CSF) (#077–04674; Wako, Osaka, Japan), recombinant human IFN-γ (#11725-HNAS; Sino Biological, Beijing, China), and recombinant mouse IFN-γ (#575304; BioLegend, San Diego, California, USA).

### M2-like phenotype macrophages (M2 macrophages) generation

The M2 macrophages were generated following previously established protocols [17, 21]. For mouse M2 macrophages, bone marrow cells were cultured for 7 days in RPMI medium with 40 ng/mL of recombinant mouse GM-CSF. After removing the GM-CSF, recombinant mouse IL-4 (20 ng/mL) was added and incubated for 48 h. Human M2 macrophages were cultivated from the THP-1 cell line. The cells were initially cultured in RPMI medium supplemented with 150 nM PMA for 24 h. After removing PMA, the medium was supplemented with human recombinant IL-4 (20 ng/mL) for an additional 48 h. Subsequently, the M2 macrophages were collected.

### Quantitative reverse transcription polymerase chain reaction (RT-PCR)

Total RNA was extracted from cancer cells using a High Pure RNA Isolation Kit (#11828665001; Roche, Basel, Switzerland) according to the manufacturer's instructions. The experimental procedure included the use of SYBR Green RT-PCR kits (#170–8892; Bio-Rad Laboratories, Hercules, CA) and the CFX96 Real-Time PCR System (Bio-Rad Laboratories) to conduct RT-PCR. Human and mouse primers were procured from Takara (Shiga, Japan) and Sigma-Aldrich, respectively. The specific primer sequences are listed in Supplementary Table S1. GAPDH was used as a reference gene to normalize mRNA expression.

### Western blotting

Following the manufacturer's instructions, the PRO-PREP Protein Extraction Solution (#17081; iNtRON Biotechnology, Seongnam, Korea) was used to obtain the proteins responsible for whole-cell lysis. Per the manufacturer's instructions, cytosolic and membrane proteins were extracted using the Mem-PER™ Plus Membrane Protein Extraction Kit (#89842; ThermoFisher Scientific, Waltham, Massachusetts, USA). In the context of sodium dodecyl sulfate–polyacrylamide gel electrophoresis, a total of 20 μg of protein were subjected to gel electrophoresis using precast gels with compositions of 4%–15% and 7.5% (Mini-PROTEAN TGX Precast Gels, #456–1084 and #456–1026, Bio-Rad Laboratories, Hercules, California, USA). The separated proteins were subsequently transferred to Trans-Blot Turbo Mini PVDF Transfer Packs (#170–4156; Bio-Rad Laboratories) using a Trans-Blot Turbo Transfer Starter System (Bio-Rad Laboratories). The membrane was incubated at 4 °C overnight with specific rabbit anti-HRH1 antibody (1:500, #13413–1-AP; Proteintech), rabbit anti-HRH1 antibody (1:500, #AHR-001; Alomone Labs, Jerusalem, Israel), mouse anti-HLA-ABC (1:1000, #ab70328; Abcam), rabbit anti-HLA-ABC (1:1000, #PA5-98355; ThermoFisher Scientific), rabbit anti-B2M (1:1000, #12851; Cell Signaling Technology, Danvers, Massachusetts, USA), rabbit anti-CANX (1:1000, #2679; Cell Signaling Technology), rabbit anti-CALR (1:1000, #12238; Cell Signaling Technology), rabbit anti-TAP1 (1:1000, #49671; Cell Signaling Technology), rabbit anti-TAP2 (1:1000, #25657; Cell Signaling Technology), rabbit anti-Methylsterol Monooxygenase 1 (1:1000, #abx027706; Abbexa, Cambridge, UK), rabbit anti-DHCR7 (1:1000, #GTX130695; Funakoshi, Tokyo, Japan), mouse anti-aSMA (1:1000, #M0851; Dako), mouse anti-IL-6 (1:1000, #ab9324; Abcam), mouse anti-β-actin (1:1000, #ab8227; Abcam), rabbit anti-β-actin (1:1000, #4970S; Cell Signaling Technology) antibodies and then probed with horseradish peroxidase-conjugated secondary antibodies (Cell Signaling Technology). The immunoblot detection procedure was conducted utilizing chemiluminescence using a ChemiDoc XRS System manufactured by Bio-Rad Laboratories (Hercules, California, USA), and *β*-actin was used as a loading control.

### Small interfering RNA silencing of MSMO1 and DHCR7

MIA PaCa-2 cells were transfected with small interfering RNA (Qiagen, Hilden, Germany) by electroporation using the Nucleofector System (Lonza, Basel, Switzerland) according to the manufacturer's instructions. In this investigation, siMSMO1 (#SI04297601), siDHCR7 (#SI00363300), and a negative control (siNC) (#1027310) were used. In subsequent experiments, the cells were used 72 h after transfection. Knockdown effectiveness was confirmed by RT-PCR and western blotting, with assessments performed 72 h after transfection.

### Small hairpin RNA transfection

One human HRH1 small hairpin (shRNA) vector (#TRCN0000011675; Sigma-Aldrich) was introduced into KP-2 cells, while three mouse HRH1 small hairpin vectors (#TRCN0000028707, #TCRN0000028668, and #TCRN0000028677; Sigma-Aldrich) were introduced into KPC cell lines (KPC-1 and KPC-2), following the manufacturer’s instructions. shRNA-transfected clones were selected using puromycin (#631305; Takara). HRH1 knockdown using shRNA was confirmed using RT-PCR and western blotting. Non-targeting shRNA (shNC) (#SHC016V-1EA, Sigma-Aldrich) was used as a control.

### BODIPY accumulation assay

CAFs (1 × 10^5^) were cultured in a 35-mm glass-bottom dish and incubated for 48 h. Alternatively, CAFs were treated with 10 μM Az and then stained with 1 mg/mL 4,4-difluoro-1,3,5,7,8-pentamethyl-4-bora-3a,4a-diaza-s-indacene (BODIPY 493/503, #D-3922; Life Technologies, Carlsbad, California, USA) and 1 mg/mL 4,6-diamidino-2-phenylindole (DAPI; Dojindo, Kumamoto, Japan) at room temperature for 1 h. The number of BODIPY-positive puncta per cell was quantified for at least 20 cells.

### Migration and invasion assays

For the migration assay, CAFs were seeded in Transwell inserts with 8-μm pores (#353097; Corning, New York, USA) at a density of 1 × 10^5^ cells per well. In the invasion assay, the cells were placed in Transwell inserts with 8 μm pores (#353097; Corning) that had been coated with 20 μg of Matrigel (#354234; BD Biosciences, Bedford, Massachusetts, USA). Following the 24-h incubation period, the samples were treated with either Az or H_2_O. Following incubation for 24 h for the migration assay or 48 h for the invasion assay, CAFs that moved to the bottom surface of either the Matrigel-coated or uncoated membrane were fixed using 70% ethanol and then stained with hematoxylin and eosin (H&E). CAFs were enumerated in five randomly selected areas at 200 × magnification.

### Cancer cells with CAFs: indirect co-culture

Human CAFs were subjected to pretreatment with Az (40 μM), whereas mouse CAFs were subjected to pretreatment with Az (20 μM), both in a 10 cm dish (#150466; thermofisher). The control group was treated with H_2_O. After 48 h, the collected CAFs (1 × 10^5^) were sown in Transwell inserts with 0.4 μm pores (#35309; Corning). Simultaneously, the cancer cells (1 × 10^5^) were seeded into 24 wells (#353504; Corning). Following a 24-h incubation period, cancer cells were subjected to indirect co-culturing with CAFs in the presence or absence of IFN-γ at concentrations of 10 or 20 ng/mL for 48 h. Subsequently, human cancer cells were harvested to assess the expression of HLA-ABC and B2M, whereas mouse cancer cells were analyzed for MHC-I expression using flow cytometry.

### Flow cytometry (FCM) analysis

For in vitro analysis, cells were removed from the culture in a 10 cm plate using a pipette. The harvested cells were incubated with FITC anti-HLA-ABC (1:100 or 1:500, #311404; BioLegend, San Diego, California, USA) or FITC anti-B2M (1:100 or 1:1000, # 316304; BioLegend) for human cancer cells (MIA PaCa-2 and KP-2), and FITC anti-H2 (1:50 or 1:100, #125508; BioLegend) for mouse cancer cells (KPC-1, KPC-2, and KPC-3). FITC IgG (#400506; BioLegend) or FITC IgG (#400108; BioLegend) was used as the control. The cells were then stained for 30 min at 4 °C. Following the removal of antibodies using phosphate-buffered saline, the cells were stained with propidium iodide (PI) (1:1000, #421301; BioLegend). Subsequently, the stained cells were analyzed using FACSVerse (BD Biosciences). The gating mechanisms used for the FCM analyses are shown in Supplementary Figure S3E. The acquired data were analyzed using FlowJo 10.5.3.

### Microarray

Total RNA was isolated from cultured cells using a High Pure RNA Isolation Kit (11,828,665,001; Roche, Basel, Switzerland) in conjunction with DNase I (Roche). RNA quality was assessed by microarray analysis using an Agilent 2200 TapeStation (Agilent Technologies, California, USA). The RNA samples were labeled and hybridized to an Agilent SurePrint G3 Human Gene Expression Microarray 8 × 60 K Ver.3.0, manufactured by Agilent Technologies. The data were analyzed using feature extraction software developed by Agilent Technologies. After identifying overlapping genes (Supplementary Table S2), Metascape was used for pathway enrichment and gene research.

### *In vivo* experiments

Female BALB/c-nu/nu and C57BL/6N mice were procured from Clea (Tokyo, Japan) and allowed a week to acclimatize. In subcutaneous implantation, a mixture of SUIT-2 cells (5 × 10^5^) and human CAF1 (5 × 10^5^) in 50 μL of DMEM was administered to the left legs of female BALB/c-nu/nu mice. We determined the proper dose of Az after preliminary experiment. One-week post-implantation, the mice (five or six per group) received intraperitoneal injections (i.p.) of either 16 mg/kg Az or H_2_O (control) three times a week for 4 weeks, with tumor volume measured weekly. For orthotopic implantation [22], a similar mixture was orthotopically injected into the tail of the pancreas in female BALB/c-nu/nu mice. One-week post-implantation, the mice (four or five per group) received either 20 mg/kg Az or H_2_O (control) via i.p. administration three times a week for 4 weeks. We also performed orthotopic co-transplantation using mouse cancer cell lines (KPC-1, luciferase-expressing KPC-2, or luciferase-expressing KPC-3) (5 × 10^5^) and mouse CAF1 (5 × 10^5^) in female C57BL/6N mice. For Az alone treatment, one-week post-implantation, mice were randomly divided into two groups (5 mice per group): one group received 20 mg/kg Az (i.p., three times a week for four weeks). For Az and the combined αPD-1 (#BE0146; BioXcell) treatment, one week post-implantation, mice were randomly divided into two groups (5 mice per group): one group received 20 mg/kg Az alone (i.p., three times a week for four weeks), and the other group received 20 mg/kg Az (i.p., three times per week for four weeks) combined with 200 μg αPD-1 (i.p., twice per week for four weeks). Alternatively, another set of mice was divided into four or six groups (4 or 5 mice per group): one group received 10 mg/kg Az alone (i.p., three times a week for four weeks), while the other groups received 10 mg/kg Az (i.p., three times per week) combined with either 100 μg or 50 μg αPD-1 (i.p., twice per week) for four or two weeks. H_2_O or IgG (#BE0090; BioXcell) was used as the control group. In the survival experiments, tumor-bearing mice were randomized into four groups of five humanely terminated mice per group based on a weight loss of 20% or manifestations of cachexia. Additionally, shNC (KPC-1, luciferase-expressing KPC-2), shHRH1 (KPC-1 (sh1, sh2), luciferase-expressing KPC-2 (sh1)), and mouse CAF1 were co-transplanted into female C57BL/6N mice, and the same number of cells were transplanted into the pancreas as described above. One week after implantation, mice were randomly divided into four groups (4 or 5 mice per group) and treated with 50 μg αPD-1, i.p., twice weekly for 2 weeks, with IgG as the control treatment group.

The IVIS spectrum imaging system (Revvity) was used to conduct measurements after intraperitoneal administration of 3 mg D-Luciferin K + Salt (#LK10000; Oz Biosciences) to mice under anesthesia. Emissions were quantified using LivingImage software version 4.3.1 (Summit Pharmaceuticals International Corporation, Tokyo, Japan). Following transplantation, the mice were euthanized, and all tumors were surgically removed and assessed. Tumor volume was calculated using the formula (3.14/6) × L × W × W, where L and W represent the largest and smallest diameters of the tumor, respectively. Peritoneal dissemination was evaluated by determining the presence or absence of intraperitoneal nodules (> 1 mm) and ascites. The assessment of liver and lung metastases included determining the presence or absence of tumors on the surface of the liver and lungs, which may be clearly distinguished from disseminated metastases.

### IHC and immunofluorescence (IF) for mouse tumor tissue

Tumor tissues were subjected to in vivo analysis by H&E staining and immunostaining. For IHC, these antibodies included mouse anti-αSMA (1:100, #M0851; Dako), rabbit anti-proliferating cell nuclear antigen (PCNA; 1:500, #ab2426; Abcam), rabbit anti-HRH1 (1:100, #13413–1-AP; Proteintech), rabbit anti-HLA-ABC (1:100, #PA5-98355; ThermoFisher Scientific), anti-rabbit CD8 (1:100, #98941; Cell Signaling Technology), and mouse anti-Granzyme B antibody (1:100, #SC-8022; Santa Cruz Biotechnology). The Picrosirius Red Staining Kit (ScyTek Laboratories, Inc.) was used to perform Sirius red staining following the manufacturer’s instructions. Positively stained areas and cells were quantified using a BZ-X Analyzer (Keyence 700). The area of αSMA and Sirius red-positive staining was quantified in five fields of view at a magnification of 100. The PCNA index was determined by calculating the percentage of PCNA-positive tumor cells relative to the total number of tumor cells. This calculation was performed for five fields of view at 100 × magnification. CD8^+^ cells were quantified by counting the number of cells in a minimum of three fields at a magnification of 100 or 200. Granzyme B expression was quantified by evaluating the number of positive cells in at least three fields viewed at 200 × magnification. Immunofluorescent labeling of the tumor tissue was performed using an anti-rabbit CD8 antibody (1:100, #98941; Cell Signaling Technology) and a mouse anti-Granzyme B antibody (1:100, #SC-8022; Santa Cruz). The secondary antibodies used in this study were EnVision System-HRP Labeled Polymer Anti-Rabbit (#K4003; Dako) or EnVision System-HRP Labeled Polymer Anti-Mouse (#K4001; Dako) for IHC and Alexa Fluor 488 conjugated anti-rabbit (A11034; Thermo Fisher) or 546 conjugated anti-mouse (A11030; Thermo Fisher) for IF. Nuclei were stained with either hematoxylin or DAPI. The percentage of Granzyme B/CD8 was determined by calculating the ratio of CD8^+^ cells expressing Granzyme B to the total number of CD8 cells multiplied by 100. This calculation was performed for at least three fields of view at a magnification of 200. The percentage of MHC-I cells was determined by calculating the ratio of MHC-I-positive cells to the total number of cells multiplied by 100, as previously described [23]. This calculation was performed for three fields of view at a magnification of 200 × .

### Statistical analysis

Statistical analyses were performed using Prism GraphPad 9.0 (San Diego, California, USA). Unless stated otherwise, data are presented as the median or mean ± standard deviation (SD). Unpaired two-tailed t-tests or Mann–Whitney test was used to analyze the data from the two independent groups. Kaplan–Meier analysis was used to assess survival outcomes, and the curves were compared using the log-rank test. Fisher's exact test and Pearson correlation coefficients were used for correlation analysis.

## Results

### HRH1 is expressed in PDAC and correlates with low HLA-ABC expression, CD8 T cell infiltration, and poor survival in human PDAC

**Fig. 1:**
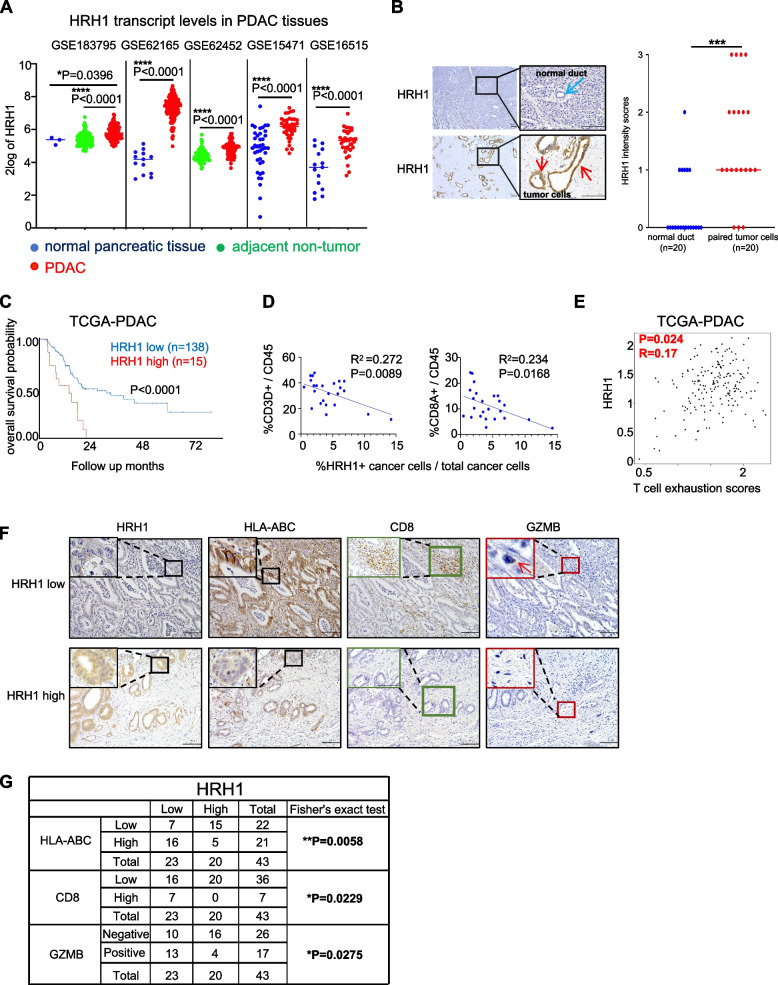
HRH1 expression in PDAC correlates with low HLA-ABC expression, CD8 T cell infiltration, and poor survival in human PDAC. **A** HRH1 mRNA expression in normal pancreatic tissue, adjacent non-tumor tissue, and PDAC tissue in public data. **B** Representative images of immunohistochemical (IHC) for HRH1 in normal pancreas and PDAC. The blue arrow indicates a normal duct. The red arrow indicates PDAC cells. Quantification of HRH1 expression intensity in normal ducts and paired tumor cells, *n* = 20. **C** Kaplan–Meier analysis of overall survival according to HRH1 expression by R2: Genomics Analysis and Visualization Platform. **D** The correlation plot of the mRNA relationships between HRH1 and CD3D^+^/CD45^+^ and CD8A^+^/CD45^+^, respectively (public data: CRA001160). **E** The correlation plot of the mRNA relationships between HRH1 and T cell exhaustion scores using GEPIA 2.0. **F** Representative images of IHC for HRH1, HLA-ABC, CD8, and GZMB in human PDAC tissues. The red arrow indicates GZMB. **G** The relationship between HRH1 protein levels and CD8, HLA-ABC, or GZMB protein levels in human PDAC tissues, *n* = 43. Scale bar = 100 µm (**B**, **F**). Median (**A**, **B**); *p < 0.05, **p < 0.01, ***p < 0.001, ****p < 0.0001. HRH1, histamine receptor H1; PDAC, Pancreatic ductal adenocarcinoma; GZMB, Granzyme B

HRH1 transcript levels were higher in cancer tissues than in normal or adjacent non-tumor tissues (Fig. 1A and Fig. S1A). Expression was confirmed at the protein level, and HRH1 expression in tumor cells was higher than that in paired normal ductal cells (Fig. 1B). Patients with high HRH1 transcript levels had poorer survival than those with low levels in the TCGA cohort (Fig. 1C). Further examination of the influence of cancer heterogeneity, such as "basal-like" type and "classical" type PDAC [24], showed high levels of HRH1 mRNA expression in both types. No difference in expression was found between the basal-like and classical types in the TCGA and GTEx analyses (Fig. S1B). We examined the relationship between HRH1 expression and immune system function. We found that single-cell RNA sequence data [25] derived from patients with PDAC showed the expression level of HRH1 in cancer cells is negatively correlated with the expression level of CD3D^+^, CD8A^+^ T cells (Fig. 1D). However, there was no relationship between HRH1 expression and other immune cell markers such as CD4^+^ T cells, Tregs, B cells, and Natural killer (NK) cells (Fig. S1C). In addition, we found that there was a positive correlation between HRH1 expression levels and T cell exhaustion scores [26] and that some exhaustion markers, such as HAVCR2 and CD39, were also positively related to HRH1 expression [27, 28] (Fig. 1E and Fig. S1D). Based on these data, immunohistochemical (IHC) was performed on our surgical specimen with HLA-ABC, CD8, and Granzyme B antibodies (Fig. 1F), and it was found that HRH1 expression was negatively correlated with HLA-ABC expression, CD8^+^ T cells, and cytotoxic CD8^+^ T cells (expressing Granzyme B) (Fig. 1G and Fig. S1E). Considering the results of TCGA dataset and IHC analysis in our sample, high HRH1 expression may be a poor prognostic factor and related with the immune responses in human PDAC.

### HRH1 antagonists enhance αPD-1 therapeutic efficacy via MHC-I upregulation

**Fig. 2:**
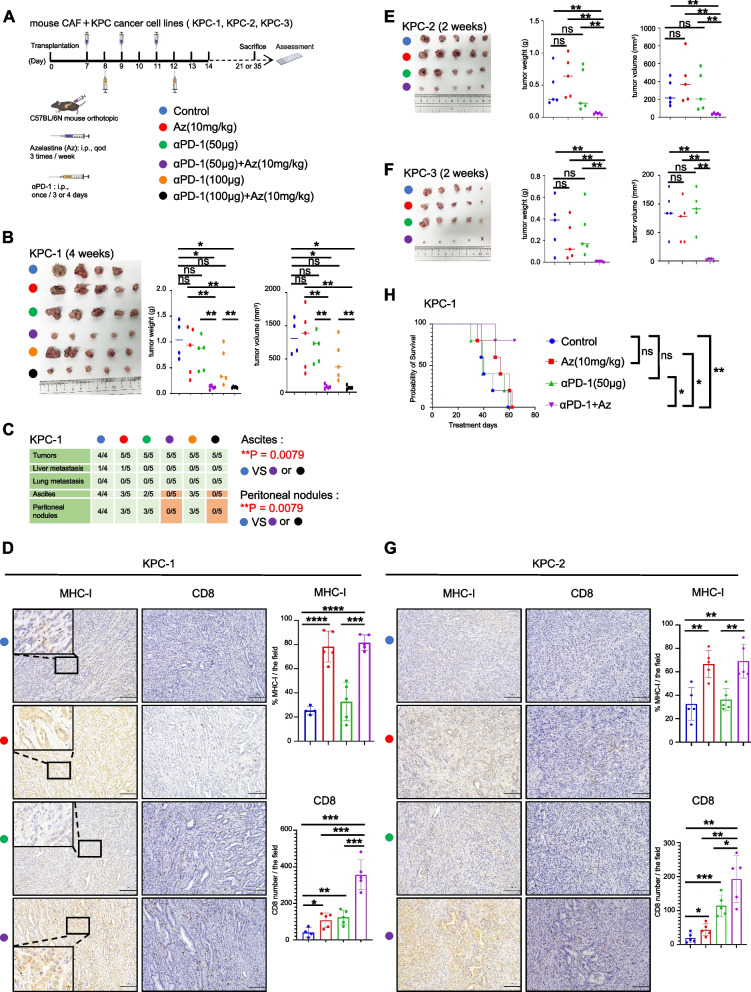
HRH1 antagonists enhance aPD-1 therapeutic efficacy via MHC-I upregulation. **A** Schema of the treatment plan for mouse cancer cell lines and the mouse CAF1 co-orthotopic transplantation model. Seven days after transplantation, treatment was started. **B**-**G** Results of the treatment in each group, (**B**, **E**, **F**) tumor picture, weight, and volume, (**C**) detection of tumor and metastasis, (**D**, **G**) representative image of IHC. Scale bar = 100 µm. (**H**) Kaplan–Meier survival curve for the co-orthotopic transplantation model. Median (**B**, **E**, **F**); error bars, mean ± SD (**D**, **G**); *p < 0.05, **p < 0.01, ***p < 0.001, ****p < 0.0001, ns, not significant. αPD-1, anti-programmed cell death protein 1; CAF1, mouse cancer-associated fibroblast 1; IHC, immunohistochemical; SD, standard deviation

To evaluate the inhibitory effect of HRH1 on the tumor microenvironment (TME) in PDAC, we investigated the effects of Az, a Food and Drug Administration (FDA) approved HRH1 antagonist, as well as its synergistic effect on αPD-1 treatment (Fig. 2A). We established mouse PDAC cell lines (KPC-1) and CAF cells (mCAF1) from primary PDAC tumors of Kras^G12D/+^, Trp53^R172H/+^, and Pdx-1-Cre (KPC) mice [29–31] and established orthotopic syngeneic PDAC models to investigate tumor growth. First, Az (20 mg/kg) was administered as monotherapy 7 days after orthotopic transplantation, and tumor growth was significantly smaller in the Az treatment group than in the non-treatment group (Fig. S2A and S2B). IHC showed a Proliferating cell nuclear antigen (PCNA) index in cancer cells, and the α-SMA expression area in CAF cells was decreased when treated with Az in tumor tissues (Fig. S2C). Subsequently, we investigated the potential synergistic effect of combining Az (20 mg/kg) with αPD-1 (200 μg). We found that combination therapy markedly reduced tumor growth and peritoneal dissemination compared to the non-treatment group (*p* = 0.0476) (Fig. S2D and S2E). Immunofluorescence (IF) showed increased CD8 and Granzyme B expression following combination therapy (Fig. S2F). The dose of each drug was reduced to confirm the synergistic effects of their combination. Az (10 mg/kg) or αPD-1 (100 µg or 50 µg) alone or in combination were administered for 4 weeks. While neither Az (10 mg/kg) nor αPD-1 (100 µg or 50 µg) suppressed tumor growth when administered as monotherapy, combination therapy of Az and αPD-1 significantly inhibited tumor growth. Strikingly, the presence of ascites and the number of peritoneal disseminations were also inhibited (*p* = 0.0079) (Fig. 2A, B and C). IHC results showed that the Az + αPD-1 combination therapy significantly increased the number of tumor-infiltrating CD8^+^ T cells and MHC-I expression in KPC-1 tumor tissue (Fig. 2D). Subsequently, we reduced the treatment duration (2 weeks) and dose and used other cancer cell lines (KPC-2 and KPC-3) derived from the primary tumor tissue of KPC mice. We observed the same results after 2 weeks of treatment (Fig. 2E-G and Fig. S2G). Finally, we discovered that the survival of tumor-bearing mice was significantly prolonged in the combination treatment group compared to that in the single treatment group (Fig. 2H). These data indicate that systematic targeting of HRH1 enhances αPD-1 therapeutic efficacy and might up-regulate MHC-I on pancreatic cancer cells in vivo.

### HRH1 inhibition of human cancer cells enhances MHC-I expression in human cancer cells

**Fig. 3:**
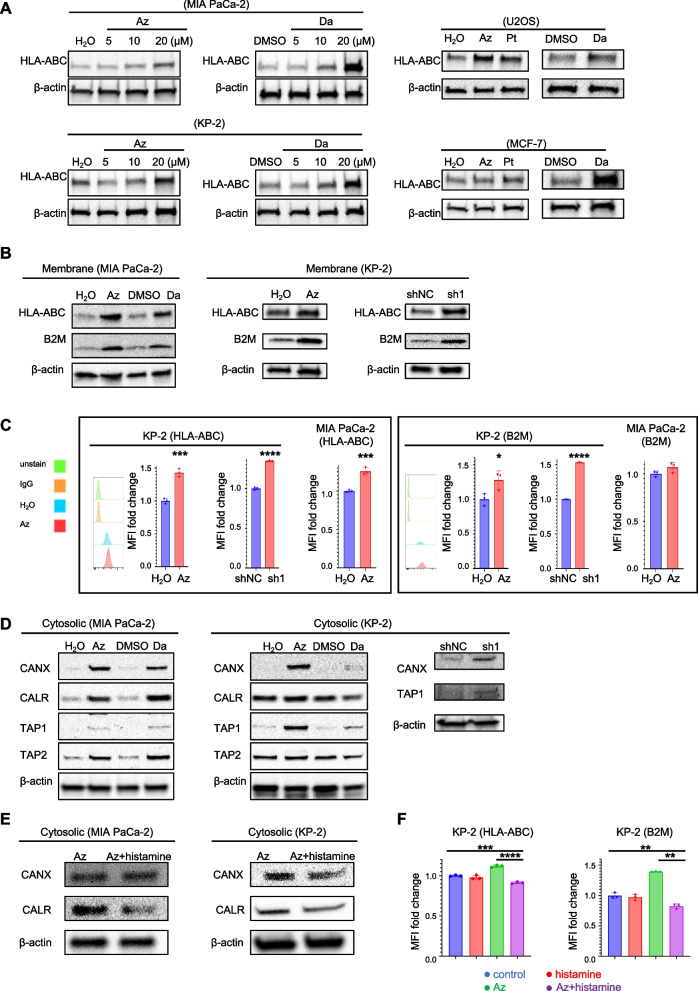
HRH1 inhibition of human cancer cells enhances MHC-I expression in human cancer cells. The expression of HLA-ABC, B2M, and HLA-related pathway proteins was determined using western blotting and FCM. **A** Various types of human cancer cell lines were treated with HRH1 antagonists for 48 h. **B** and **C** HLA-ABC or B2M expression of MIA PaCa-2 or KP-2 treated with HRH1 antagonists (20 µM) for 48 h or shHRH1 (sh1). **D** Protein expression of MIA PaCa-2 or KP-2 treated with HRH1 antagonists (20 µM) for 48 h or KP-2 shNC and shHRH1 (sh1). **E** Protein expression of MIA PaCa-2 treated with Az (20 µM) or combined with histamine (10 µM) for 48 h, (**F**) KP-2 treated with Az (20 µM) or combined with histamine (5 µM) for 24 h. Error bars, mean ± SD (**C**, **F**); *p < 0.05, **p < 0.01, ***p < 0.001, ****p < 0.0001. MHC, major histocompatibility complex; HLA, Human Leukocyte Antigen; HLA-ABC, HLA Class 1 ABC; B2M, Beta-2 microglobulin

To further evaluate the effect of HRH1 inhibitors on MHC-I expression, HRH1 antagonists, including Az, Da, and Pt, were used in in vitro experiments. HLA-ABC expression in pancreatic cancer cell lines was increased by HRH1 antagonists (Fig. 3A). We confirmed this effect using other cancer cell lines, such as U2OS (an osteosarcoma cell line) and MCF-7 (a breast cancer cell line) and obtained the same findings. Among the pancreatic cancer cell lines studied, KP-2 exhibited the highest level of HRH1 expression (Fig. S3A and S3B). Subsequently, we generated KP-2 cells with a stable HRH1 knockdown by evaluating HRH1 mRNA and protein expression (Fig. S3C and S3D). Next, we investigated HLA-ABC and B2M expression on cell membranes by western blotting and found that the expression was increased by Az or Da treatment or HRH1 knockdown (Fig. 3B). We confirmed these results using flow cytometry (Fig. 3C and Fig. S3E). These results suggest that HRH1 depletion or pharmacologic HRH1 inhibition in tumor cells promotes MHC-I expression. Next, we investigated the expression of genes relevant to the MHC-I pathway to further study MHC-I upregulation. MHC-I-related pathway genes, such as CANX, CALR, TAP1, and TAP2, were the same at the whole lysis level; however, their protein expression levels differed at the cytosolic lysis in each cell line, suggesting the difference of the translocation or release from organelles such as ER. (Fig. S3F). The protein expression levels of CANX, CALR, TAP1, and TAP2 were increased in response to HRH1 antagonist treatment or HRH1 deletion (Fig. 3D). In addition to the increase in HLA-ABC, we also observed an increase in CANX, TAP1, and TAP2 transcript levels in response to Az treatment or HRH1 depletion (Fig. S3G). These effects were reversed by treatment with histamine (Fig. 3E and F and Fig. S3H). These results indicate that HRH1 affects the transcription levels of MHC-I pathway-related genes.

### Cholesterol biosynthesis pathway is related to MHC-I expression and simvastatin-reduced Az or HRH1 knockdown-related MHC-I upregulation

**Fig. 4:**
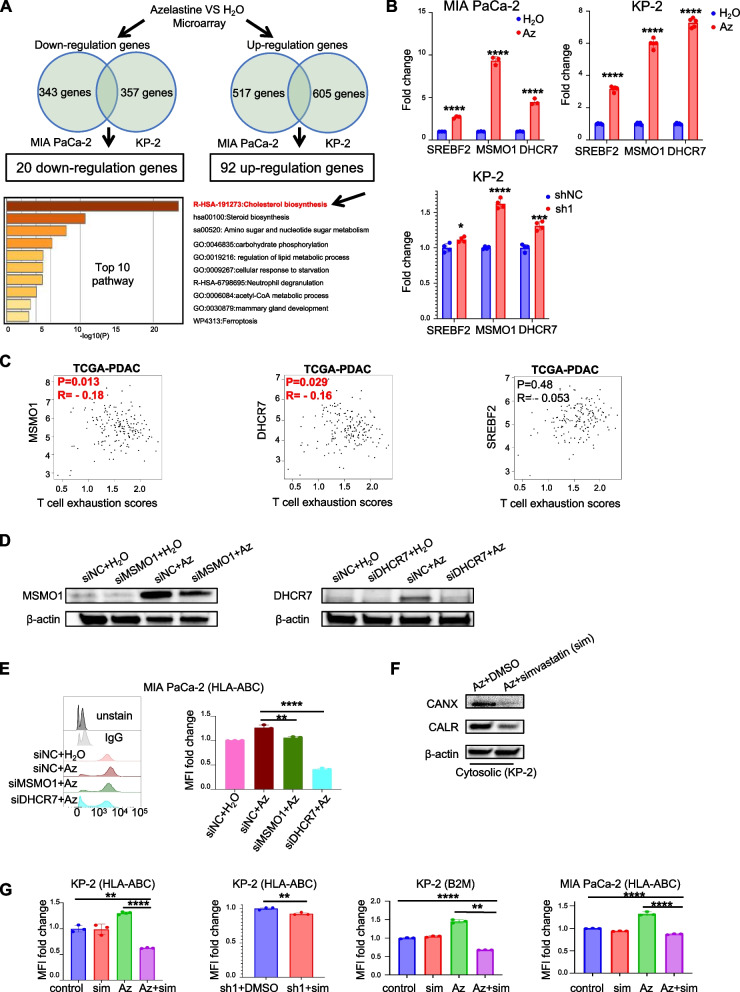
Cholesterol biosynthesis pathway is related to MHC-I expression and simvastatin-reduced azelastine or HRH1 knockdown-related MHC-I upregulation. **A** Demographics of microarray analysis of MIA PaCa-2 or KP-2 treated with Az (20 µM) or H_2_O for 48 h (upper), and the Metascape analysis pathway-enriched upregulated genes (lower). **B** RT-PCR for cholesterol biosynthesis-related gene expression in MIA PaCa-2 or KP-2 treated with Az (20 µM) for 48 h or KP-2 shHRH1 (sh1), *n* ≥ 3 per group. **C** The correlation plot shows cholesterol biosynthesis-related genes between T cell exhaustion scores by GEPIA 2.0. **D** MSMO1 or DHCR7 protein expression in whole lysis of siDHCR7 or siMSMO1, or Az (20 µM) or combination. **E** MFI of HLA-ABC expression using FCM for Az (20 µM) or combination, *n* = 3 per group. **F** CANX and CALR protein expression in KP-2 treated by Az (20 µM) or combined with simvastatin (10 µM) for 48 h. **G** MFI of HLA-ABC and B2M using FCM for MIA PaCa-2 or KP-2 treated alone or in combination, *n* ≥ 3 per group. Error bars, mean ± SD (**B**, **E**, **G**); *p < 0.05, **p < 0.01, ***p < 0.001, ****p < 0.0001. MHC-I, major histocompatibility complex class I; RT-PCR, quantitative reverse transcription polymerase chain reaction; MFI, median fluorescence intensity; FCM, Flow cytometry

To further explore how HRH1 inhibition controls the antigen presentation pathway, we performed a gene expression microarray analysis. Twenty down-regulated and 92 upregulated genes were identified. These genes were detected using Metascape [32] pathway analysis, and the cholesterol biosynthesis pathway was identified as the pathway with the highest score in the microarray analysis (Fig. 4A). We focused on representative genes involved in activating the cholesterol biosynthesis pathway, such as sterol regulatory element-binding protein 2 (SREBF2), methylsterol monooxygenase 1 (MSMO1), and 7-Dehydrocholesterol Reductase (DHCR7) [33, 34], which were included in the 92 up-regulated genes. Genetic or pharmacological inhibition of HRH1 increased the expression of these genes at the transcriptional level (Fig. 4B). We explored whether these three genes were associated with T-cell function. Using GEPIA 2.0, we found that MSMO1 and DHCR7 were negatively correlated with T cell exhaustion scores and T cell exhaust-related genes (Fig. 4C and Fig. S4A). Moreover, we created MSMO1 and DHCR7 knockdown cancer cells and discovered that MSMO1 or DHCR7 inhibition rescued the Az-induced increase in HLA-ABC (Fig. 4D and E and Fig. S4B). Previous reports have suggested that cholesterol is crucial in facilitating antigen binding to MHC class I [35]. To investigate whether the inhibition of HRH1 elevates HLA-ABC or B2M due to the activation of the cholesterol biosynthesis pathway, we used simvastatin as an inhibitor of cholesterol biosynthesis [36]. Simvastatin treatment inhibited the Az-induced increase in CANX and CALR levels (Fig. 4F). Next, we examined the expression levels of HLA-ABC and B2M by flow cytometry, which showed that simvastatin-reduced HLA-ABC and B2M upregulation in the cell membrane by HRH1 inhibition (Fig. 4G). These data suggest that genes related to cholesterol biosynthesis are involved in antigen presentation via HRH1 inhibition.

### Azelastine induces quiescent-like fibroblasts to reverse the antigen presentation of cancer cells

**Fig. 5:**
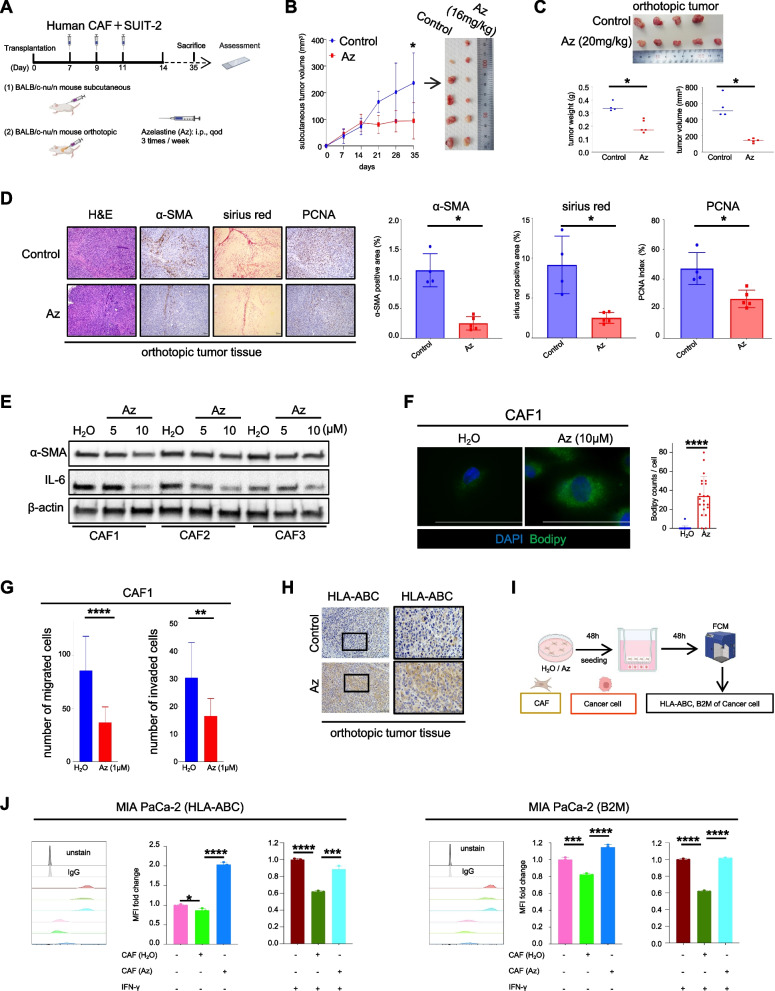
Azelastine induces quiescent-like fibroblasts to reverse the antigen presentation of cancer cells. **A** Schema of SUIT-2 and CAF1 subcutaneous or orthotopic models for 4 weeks of Az treatment, (**B** and **C**) tumor picture, volume and/or weight, (**D**) representative image of H&E, sirius red, IHC of α-SMA and PCNA. **E** α-SMA and IL-6 protein expression in whole lysis of human CAF1, CAF2, and CAF3. **F** Representative image of CAF1 stained with BODIPY. **G** The effect of treatment on migration and invasion of CAF1. **H** Representative image of the IHC for HLA-ABC. **I** Schema of MIA PaCa-2 with CAFs indirect co-culture. **J** MFI of HLA-ABC or B2M of MIA PaCa-2 detected by FCM, *n* = 3 per group. Scale bar = 100 µm (**D**, **F**, **H**). Median (**C**); error bars, mean ± SD (**B**, **D**, **F**, **G**, **J**); *p < 0.05, **p < 0.01, ***p < 0.001, ****p < 0.0001. CAF1, human cancer-associated fibroblast 1; Az, Azelastine; H&E, hematoxylin and eosin; α-SMA, α-smooth muscle actin; PCNA, Proliferating cell nuclear antigen; IL-6, Interleukin 6

In addition to the increase of MHC-I expression in cancer cells, we also found that α-SMA expression, one of the CAF activation markers, decreased in vivo (Fig. S2C). We established subcutaneous and orthotopic models in nude mice using a human pancreatic cancer cell line and CAFs (Fig. 5A) because our previous reports showed that inhibiting CAF suppressed tumor progression by disrupting the interaction between cancer cells and activated CAF [22, 37–39]. Az was administered 1 week after transplantation, and tumor size was measured weekly. Tumor growth was suppressed within 4 weeks of Az treatment in both the subcutaneous (Fig. 5B) and orthotopic (Fig. 5C) models. We also observed a decrease in the PCNA index in cancer cells and a reduction in α-SMA and sirius red staining (Fig. 5D). To investigate the role of Az in targeting CAF activation in vitro, CAFs were treated with Az. α-SMA and IL-6 expression decreased depending on Az dosage and induced bodipy accumulation by Az (Fig. 5E and F and Fig. S5A). Moreover, Az treatment suppressed CAF migration and invasion (Fig. 5G and Fig. S5B). In addition, Az treatment increased HLA-ABC expression in cancer cells in the orthotopic model (Fig. 5H). To understand whether the inhibition of CAF activation could enhance the antigen presentation ability of cancer cells, CAFs were treated with either H_2_O or Az. Subsequently, CAFs were collected for indirect co-culture with cancer cells. After a 48-h co-culture period, flow cytometry (FCM) was used to determine the expression of HLA-ABC and B2M in human cancer cells (Fig. 5I). The findings of this study indicated that activated human CAFs decreased HLA-ABC and B2M expression in human cancer cells, which was reversed by Az-mediated suppression of CAF activation (Fig. 5J). We also found the same results in mouse cancer cells (Fig. S5C and S5D). These results suggest that Az also has the potential to enhance HLA-ABC expression in cancer cells by inducing quiescent CAFs.

### Cancer cell-specific HRH1 depletion enhances αPD-1 therapeutic efficacy in mice

**Fig. 6:**
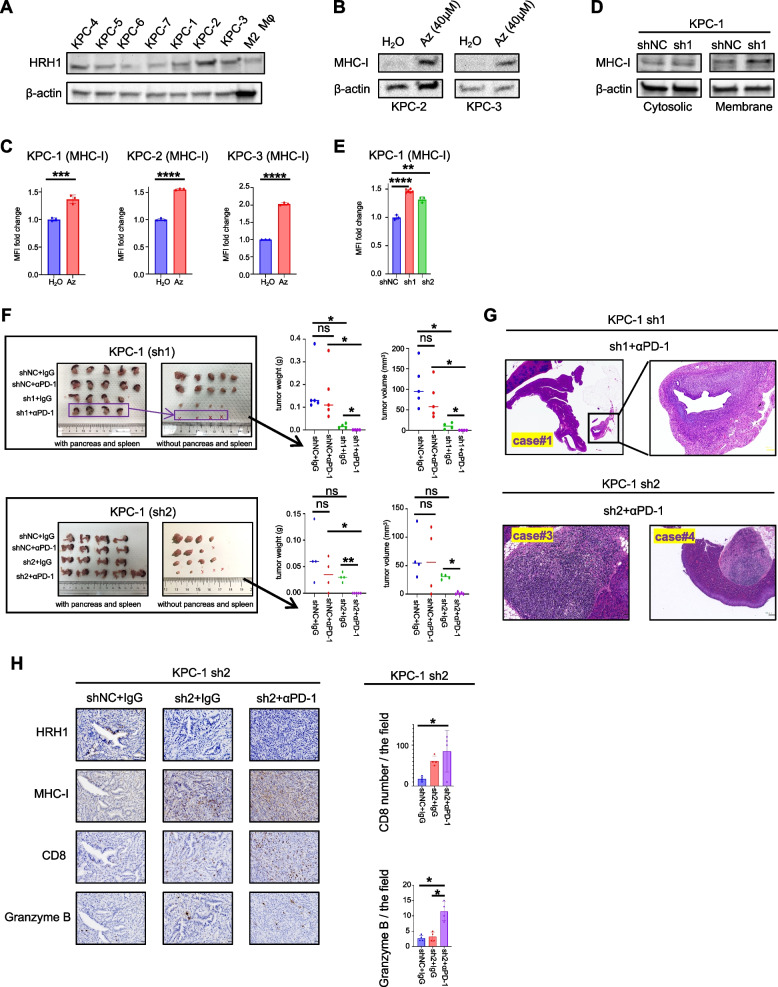
Cancer cell-specific HRH1 depletion enhances αPD-1 therapeutic efficacy in mice. **A** HRH1 protein expression in mouse whole lysis of cancer cell lines (KPC-1 to KPC-7) and M2 macrophages. **B** MHC-I protein expression in whole lysis of KPC-2 and KPC-3 treated with Az or H_2_O for 48 h. **C** MFI of MHC-I using FCM for KPC-1, KPC-2, and KPC-3 treated with Az (40 µM) or H_2_O for 48 h, *n* = 3 per group. **D** MHC-I protein expression in KPC-1 shHRH1 (sh1) and shNC. **E** MFI of MHC-I using FCM for KPC-1 shHRH1 (sh1, sh2) and shNC, *n* ≥ 3 per group. **F**–**H** The orthotopic co-transplanted syngeneic tumors (KPC-1 shNC, shHRH1 (sh1 or sh2), and mouse CAF1) for treatment of 2 weeks, (**F**) tumor picture, volume, and weight, (**G**) H&E of KPC-1 (**H**) representative image of IHC. Scale bar = 20 µm Median (**F**); error bars, mean ± SD (**C**, **E**, **H**); *p < 0.05, **p < 0.01, ***p < 0.001, ****p < 0.0001

Our results suggest that the systemic pharmacological blockade of HRH1 may play a multifunctional role in enhancing antigen presentation in cancer cells. Therefore, we investigated whether genetic deletion of HRH1 in cancer cells can enhance the therapeutic efficacy of αPD-1 treatment. We examined mRNA expression levels of HRH1 in KPC-derived cancer cell lines (Fig. S6A). To further validate the expression of HRH1, we measured the protein levels in cancer cell lines via western blotting. We used previously reported M2-like macrophages as positive controls for HRH1. Mouse cancer cells expressed various levels of HRH1 protein (Fig. 6A). MHC-I expression in mouse cancer cells increased after Az treatment (Fig. 6B and C). Furthermore, we established HRH1 knockdown mouse cell lines and determined their knockdown efficiency using PCR and western blotting (Fig. S6B-S6D). We observed increased MHC-I expression on the membranes following HRH1 knockdown (Fig. 6D and E). Next, we orthotopically transplanted CAF1 and HRH1 knockdown cancer cells into C57BL/6N mice. After 2 weeks of treatment with aPD-1 (50 µg), tumor growth was significantly suppressed in the HRH1 knockdown group, and some of the tumors in the HRH1 knockdown + αPD-1 group were not detected macroscopically (Fig. 6F and Fig. S6E and S6F). Subsequently, we performed hematoxylin and eosin (H&E) staining and found microscopically detectable small cancer cells in macroscopically undetectable specimens’ case #1 of KPC-1 in sh1 + αPD-1 group; case #3 and case #4 of KPC-1 in sh2 + αPD-1 group, suggesting tumor growth was truly suppressed (Fig. 6G**)**. Moreover, immunohistochemical analysis revealed in the HRH1 knockdown with αPD-1 group (sh2 + αPD-1), an increase in Granzyme B expression and CD8 T cell infiltration compared to other groups was observed within the tumor (Fig. 6H).

## Discussion

Pancreatic cancer is considered a non-immunogenic “cold” tumor, characterized by low CD8^+^ T cell infiltration and resistance to immune checkpoint inhibitors [40]. Converting this “cold” state to a “hot” one is a promising approach to enhancing the disease’s prognosis. This study demonstrated that anti-PD1 treatment combined with pharmacological or genetic HRH1 inhibition significantly enhanced tumor growth suppression in PDAC mouse models. This enhancement was characterized by increased MHC-I expression in cancer cells and the promotion of CD8^+^ T cell infiltration in tumor tissues. Although HRH1 has been targeted in cancer therapy with favorable outcomes [41, 42], only a few studies have delved into its relationship with the immune system [16, 17]. Recently, HRH1 was reported to shift macrophages toward an immunosuppressive M2 phenotype and suppress CD8^+^ T cell function, and additional treatment with antihistamines was found to amplify the effect of anti-PD1 antibodies in melanoma [17]. Although cancers typically respond well to ICB [43, 44], our study indicated that the sole use of anti-PD1 did not affect tumor growth. Contrastingly, additional HRH1 antagonist treatment resulted in drastic effects, including a complete response in some mice. These findings suggest a transformation from ‘cold’ to ‘hot’ tumor status. This raises a “chicken and egg” question regarding the upregulation of the MHC-I pathway by IFN-γ secreted from CD8^+^ T cells [45]. Despite this, our findings indicated that HRH1 inhibition independently increases MHC-I expression, as demonstrated by enhanced expression even without CD8^+^ T cells in vitro.

Microarray analysis revealed that the cholesterol biosynthesis pathway was significantly increased compared to other pathways in the Az treatment group. We validated the expression of the cholesterol synthesis genes SREBF2 (SREBP2), MSMO1, and DHCR7, induced by pharmacological or genetic HRH1 inhibition. The importance of cholesterol metabolism in cancer immunotherapy has recently been highlighted. However, reports on its role in regulating immunotherapies are conflicting [46]. PSCK9 inhibition increases MHC-I expression and enhances immune checkpoint therapy in cancer. Although these results seem to conflict with ours, the mechanism is independent of cholesterol regulation, relying on direct interaction with MHC-I and reducing lysosomal degradation [11]. Generally, inhibiting cholesterol synthesis promotes autophagy [47, 48]. The mechanisms underlying MHC-I expression in our study may involve: 1) Direct interaction of genes (SREBF2, MSMO1, and DHCR7) and MHC-I signaling genes (TAP1 and TAP2) in the endoplasmic reticulum (ER). 2) Cholesterol’s role in inhibiting autophagy [9]. 3) Increased cholesterol levels’ effect on MHC-I signaling. In this study, an increase in MHC-I expression was tumor specific and not observed in normal organs. Although cholesterol metabolism regulates the antitumor immune response, its complex mechanism of action requires further investigation to identify potential targets.

We hypothesized that CAF also affects the infiltration of T cells through the transformation of MHC-I expression in cancer cells. Previous studies have highlighted CAFs and matrix components as primary contributors to ICB resistance in PDAC [49–51]. In our in vitro experiment, co-culture with CAFs led to significantly reduced MHC-I expression in cancer cells, a decrease reversed by HRH1-antagonist-pretreated CAFs, suggesting that CAFs diminish MHC-I expression. While direct interaction between CAFs and MHC-I expression in cancer cells has not been reported, cytokines secreted by CAFs may play a role in this process. In addition, tumor growth inhibition was observed in T cell deficient mice treated with HRH1 antagonists, marked by decreased αSMA and Sirius red expression, indicating decreased levels of CAF activation. These findings were confirmed through in vitro experiments, such as decreased levels of IL-6 expression in CAFs after HRH1 antagonist treatment [37, 52, 53]. These findings suggest Az also directly inhibited CAF activation and inhibited tumor growth by decreasing tumor-stromal interaction. Although mechanisms underlying CAF reprogramming by HRH1 antagonists have not yet been investigated, our previous work reported autophagy as a key regulator of CAF activation. Further investigations are required to elucidate this mechanism.

## Conclusions

In summary, HRH1 inhibition significantly impacts MHC-I expression in cancer cells, potentially altering the “cold” to “hot” immunogenic status in PDAC. Combined use of an HRH1 antagonist and anti-PD-1 antibody notably suppressed PDAC growth in mouse models, indicating the potential to improve prognosis in this cancer type. With HRH1 widely acknowledged as an allergy regulator and its antagonists safely used, clinical targeting of HRH1 signaling is poised to improve the prognosis of PDAC.

### Supplementary Information

**Additional file 1: Supplementary Fig. S1. **(A, B) The HRH1 mRNA expression levels were analyzed by GEPIA 2.0. (A) Normal pancreas and PDAC. (B) Normal pancreas and basal types of PDAC, normal pancreas and classical types of PDAC. (C) The correlation plot of the mRNA relationship between HRH1 expression and other immune cell markers by Fisher's exact test. (D) The correlation plot of the mRNA relationship between HRH1 expression and T cell exhaustion markers by GEPIA 2.0. (E) The relationships between the number of CD8^+^ or Granzyme B^+^ cells and HRH1 protein expression in PDAC tissues, *n* = 43; median (E); *p<0.05, **p<0.01. PDAC, Pancreatic ductal adenocarcinoma.

**Additional file 2: Supplementary Fig. S2. **Orthotopically transplanted tumors of KPC-1 cells after 4 weeks of treatment with control (H_2_O and IgG), azelastine (Az), and combination therapy. (A, D) Tumor weight and volume. (B, E) Detection of tumors and metastases. (C, G) Representative image of the IHC. (F) Immunofluorescence (IF) for CD8 (green) and Granzyme B (red). Scale bar, 50 µm (G), 100 µm (C, F). Median (A, D); error bars, mean ± SD (C, F, G); *p<0.05, **p<0.01, ****p<0.0001. SD, standard deviation.

**Additional file 3: Supplementary Fig. S3**. (A) RT-PCR for HRH1 mRNA expression in human pancreatic cancer cell lines, *n* = 4 per group. (B) HRH1 expression in the whole lysis of human pancreatic cancer cell lines. (C) RT-PCR for HRH1 mRNA expression in KP-2 shNC and shHRH1 (sh1), *n*=4 per group. (D) HRH1 expression in the whole lysis of KP-2 shNC and shHRH1 (sh1). (E) FCM gating strategy for HLA-ABC or B2M of human pancreatic cancer cell lines (MIA-PaCa2). (F) The expression of HLA-related proteins in human pancreatic cancer cell lines. (G) RT-PCR for MHC-I-related gene expression in MIA PaCa-2 or KP-2 treated with Az (20 µM) for 48 h or KP-2 shNC and shHRH1 (sh1), n ≥ 3 per group. (H) MFI of HLA-ABC using FCM for MIA PaCa-2 treated with Az (20 µM), histamine (10 µM), or combination for 48 h, *n* = 3 per group. Error bars, mean ± SD (A,C,G,H); *p<0.05, **p<0.01, ***p<0.001, ****p<0.0001. RT-PCR, quantitative reverse transcription polymerase chain reaction; FCM, Flow cytometry; HLA, Human Leukocyte Antigen; HLA-ABC, HLA Class 1 ABC; B2M, Beta-2 microglobulin; MHC, major histocompatibility complex; MFI, median fluorescence intensity.

**Additional file 4: Supplementary Fig. S4**. (A) The correlation plot shows that cholesterol biosynthesis-related genes are associated with T cell exhaustion markers by GEPIA 2.0. (B) RT-PCR for siDHCR7, siMSMO1, Az (20 µM), or combination. error bars, mean ± SD (B); *p<0.05, ***p<0.001.

**Additional file 5: Supplementary Fig. S5**. (A) Representative image of CAF2 stained with BODIPY. Scale bar = 100 µm. (B) The effect of treatment on migration and invasion of CAF2. (C) α-SMA protein expression in whole lysis of mouse CAF1. (D) FCM of MHC-I expression in each group. Error bars, mean ± SD (A,B); **p<0.01, ****p<0.0001. CAF2, human cancer-associated fibroblast 2; α-SMA, α-smooth muscle actin; CAF1, cancer-associated fibroblast 1; MHC-I, major histocompatibility complex class I.

**Additional file 6: Supplementary Fig. S6**. (A) HRH1 mRNA expression in mouse pancreatic cancer cell lines (KPC-1 to KPC-7), *n* = 5 per group. (B) HRH1 mRNA expression in KPC-1 shNC and shHRH1 (sh1, sh2), *n* = 4 per group. (C) HRH1 and MHC-I protein expression in whole lysis of KPC-1 shNC and shHRH1 (sh1, sh2). (D) KPC-2 shNC and shHRH1 (sh1, sh2, sh3), *n* = 4 per group. (E, F) The orthotopic co-transplanted syngeneic tumors (luciferase-expressing KPC-2 shNC and shHRH1 (sh1)) for treatment of 2 weeks; (E) bioluminescent images of KPC-2 tumors; (F) tumor picture, volume, and weight. Median (F); error bars, mean ± SD (A,B,D); *p<0.05, **p<0.01. HRH1, histamine receptor H1; MHC-I, major histocompatibility complex class I; H&E, hematoxylin and eosin.

**Additional file 7: Supplementary Table S1.** Primers used for RT-PCR.

**Additional file 8: Supplementary Table S2.** Overlapping up-regulated genes of human cell lines.

## Data Availability

All the data produced or analyzed during this investigation are included in this article or the Supplementary file. The microarray data have been stored in the Gene Expression Omnibus. The assigned identifier is GSE251639.

## Abbreviations

HRH1: Histamine receptor H1
PDAC: Pancreatic ductal adenocarcinoma
GZMB: Granzyme B
αPD-1: Anti-programmed cell death protein 1
CAF: Cancer-associated fibroblast
IHC: Immunohistochemical
SD: Standard deviation
MHC-I: Major histocompatibility complex class I
HLA-ABC: Human Leukocyte Antigen Class 1 ABC
B2M: Beta-2 microglobulin
RT-PCR: Quantitative reverse transcription polymerase chain reaction
MFI: Median fluorescence intensity
FCM: Flow cytometry
Az: Azelastine
H&E: Hematoxylin and eosin
IHC: Immunohistochemical
α-SMA: α-Smooth muscle actin
PCNA: Proliferating cell nuclear antigen
IL-6: Interleukin 6
KPC: Kras^G12D/+^, Trp53^R172H/+^, and Pdx-1-Cre
