# Supplementary figures and images for: Blockade of histamine receptor H1 augments immune checkpoint therapy by enhancing MHC-I expression in pancreatic cancer cells
Source: J Exp Clin Cancer Res. 2024 May 8;43:138. doi: 10.1186/s13046-024-03060-5 (PMC11077718; doi:10.1186/s13046-024-03060-5)

**Supplementary Figure 1**

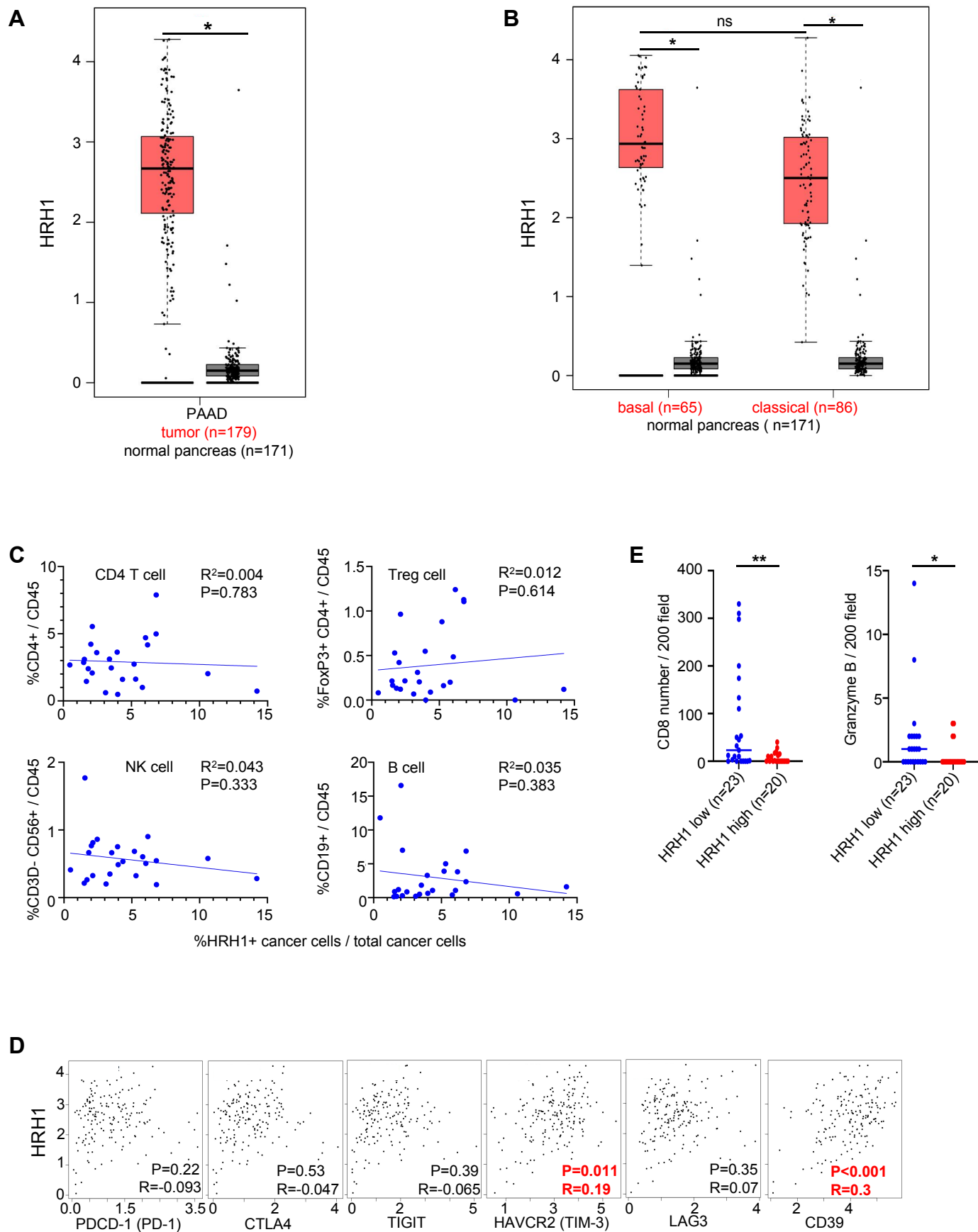

Supplement: Supplementary file 1 — Additional file 1: Supplementary Fig. S1. (A, B) The HRH1 mRNA expression levels were analyzed by GEPIA 2.0. (A) Normal pancreas and PDAC. (B) Normal pancreas and basal types of PDAC, normal pancreas and classical types of PDAC. (C) The correlation plot of the mRNA relationship between HRH1 expression and other immune cell markers by Fisher's exact test. (D) The correlation plot of the mRNA relationship between HRH1 expression and T cell exhaustion markers by GEPIA 2.0. (E) The relationships between the number of CD8+ or Granzyme B+ cells and HRH1 protein expression in PDAC tissues, n = 43; median (E); *p<0.05, **p<0.01. PDAC, Pancreatic ductal adenocarcinoma. [file 13046_2024_3060_MOESM1_ESM.pdf]

Supplementary Figure 2

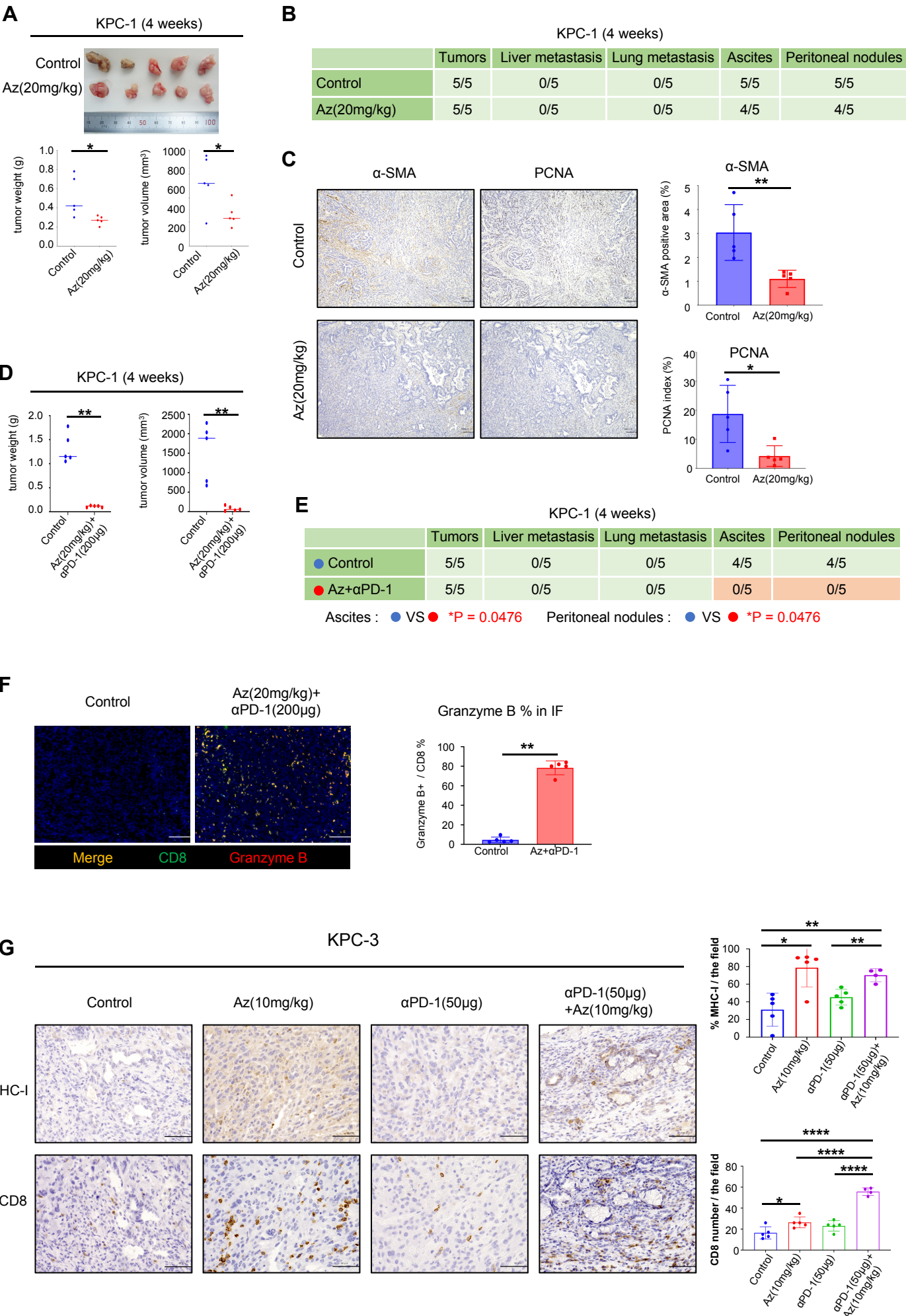

Supplement: Supplementary file 2 — Additional file 2: Supplementary Fig. S2. Orthotopically transplanted tumors of KPC-1 cells after 4 weeks of treatment with control (H2O and IgG), azelastine (Az), and combination therapy. (A, D) Tumor weight and volume. (B, E) Detection of tumors and metastases. (C, G) Representative image of the IHC. (F) Immunofluorescence (IF) for CD8 (green) and Granzyme B (red). Scale bar, 50 µm (G), 100 µm (C, F). Median (A, D); error bars, mean ± SD (C, F, G); *p<0.05, **p<0.01, ****p<0.0001. SD, standard deviation. [file 13046_2024_3060_MOESM2_ESM.pdf]

# Supplementary Figure 3

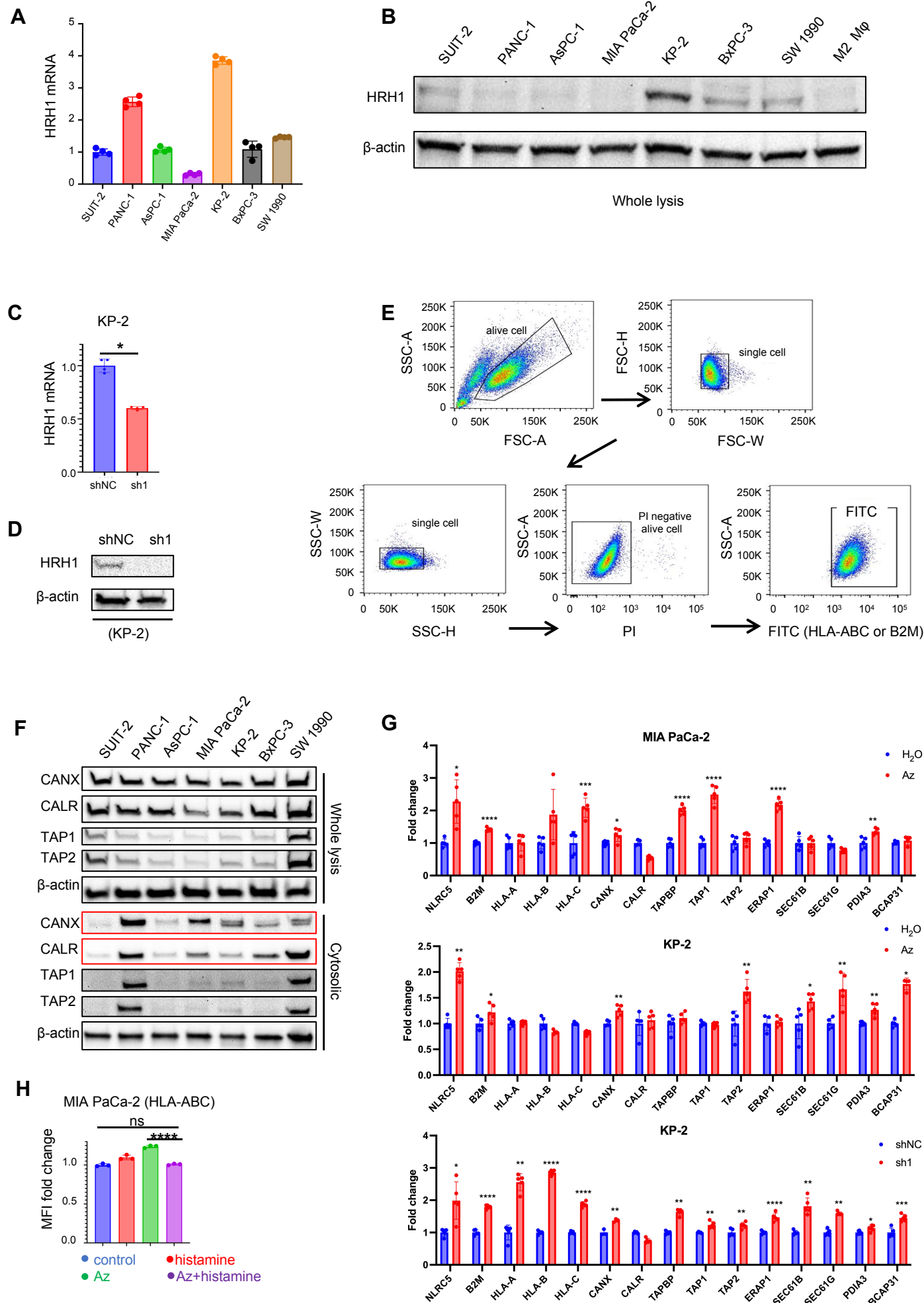

Supplement: Supplementary file 3 — Additional file 3: Supplementary Fig. S3. (A) RT-PCR for HRH1 mRNA expression in human pancreatic cancer cell lines, n = 4 per group. (B) HRH1 expression in the whole lysis of human pancreatic cancer cell lines. (C) RT-PCR for HRH1 mRNA expression in KP-2 shNC and shHRH1 (sh1), n=4 per group. (D) HRH1 expression in the whole lysis of KP-2 shNC and shHRH1 (sh1). (E) FCM gating strategy for HLA-ABC or B2M of human pancreatic cancer cell lines (MIA-PaCa2). (F) The expression of HLA-related proteins in human pancreatic cancer cell lines. (G) RT-PCR for MHC-I-related gene expression in MIA PaCa-2 or KP-2 treated with Az (20 µM) for 48 h or KP-2 shNC and shHRH1 (sh1), n ≥ 3 per group. (H) MFI of HLA-ABC using FCM for MIA PaCa-2 treated with Az (20 µM), histamine (10 µM), or combination for 48 h, n = 3 per group. Error bars, mean ± SD (A,C,G,H); *p<0.05, **p<0.01, ***p<0.001, ****p<0.0001. RT-PCR, quantitative reverse transcription polymerase chain reaction; FCM, Flow cytometry; HLA, Human Leukocyte Antigen; HLA-ABC, HLA Class 1 ABC; B2M, Beta-2 microglobulin; MHC, major histocompatibility complex; MFI, median fluorescence intensity. [file 13046_2024_3060_MOESM3_ESM.pdf]

Supplementary Figure 4

A

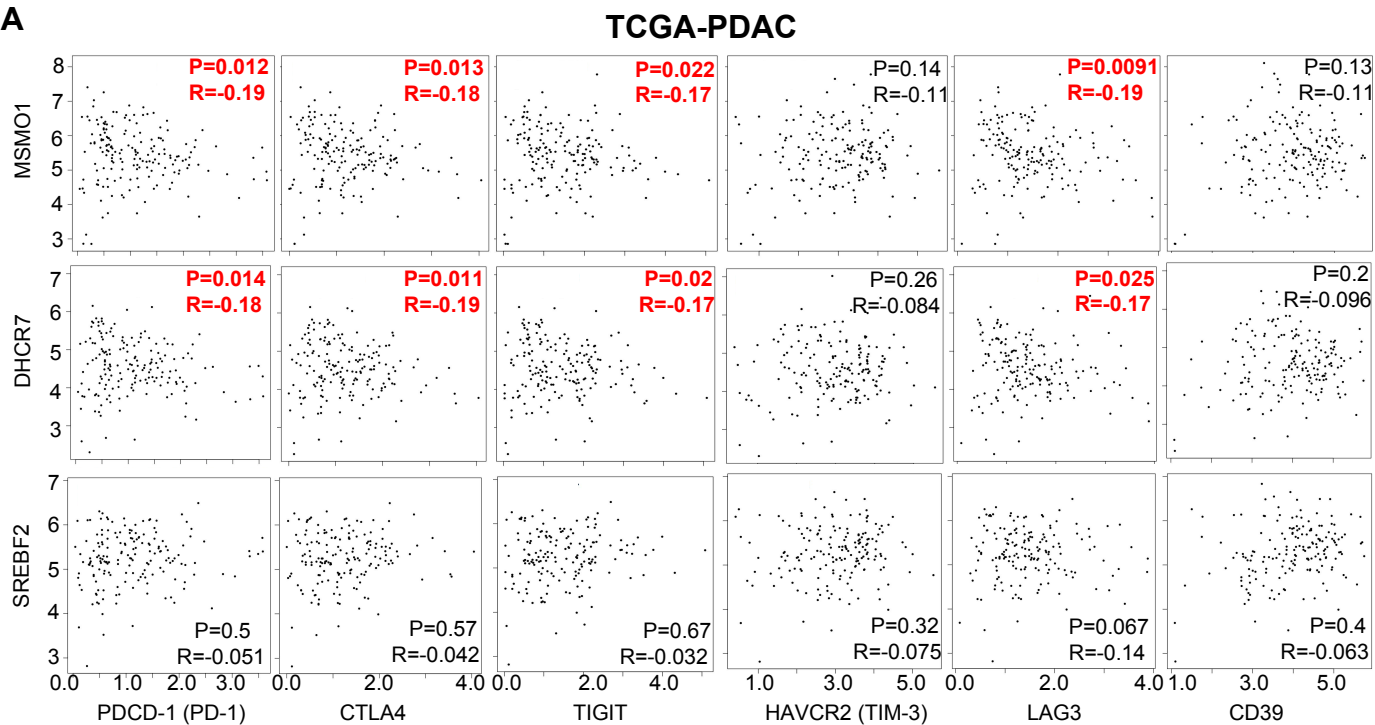

B

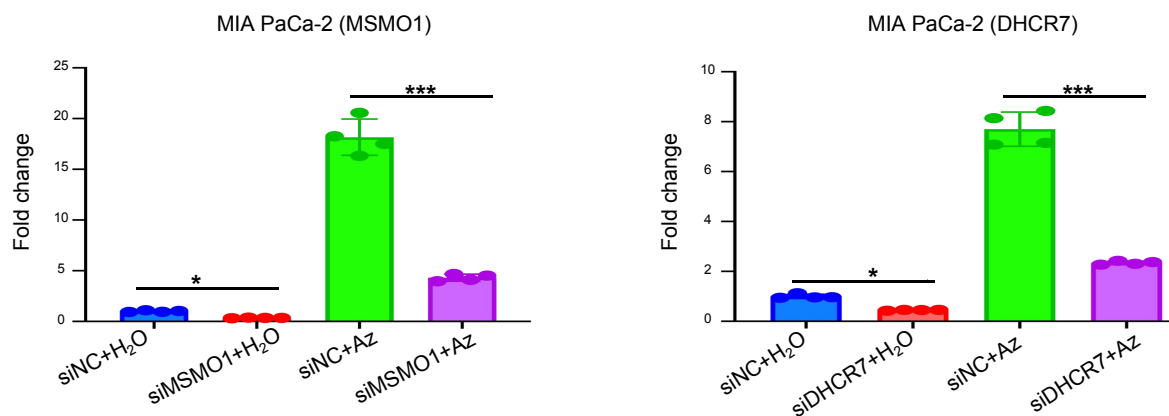

Supplement: Supplementary file 4 — Additional file 4: Supplementary Fig. S4. (A) The correlation plot shows that cholesterol biosynthesis-related genes are associated with T cell exhaustion markers by GEPIA 2.0. (B) RT-PCR for siDHCR7, siMSMO1, Az (20 µM), or combination. error bars, mean ± SD (B); *p<0.05, ***p<0.001. [file 13046_2024_3060_MOESM4_ESM.pdf]

Supplementary Figure 5

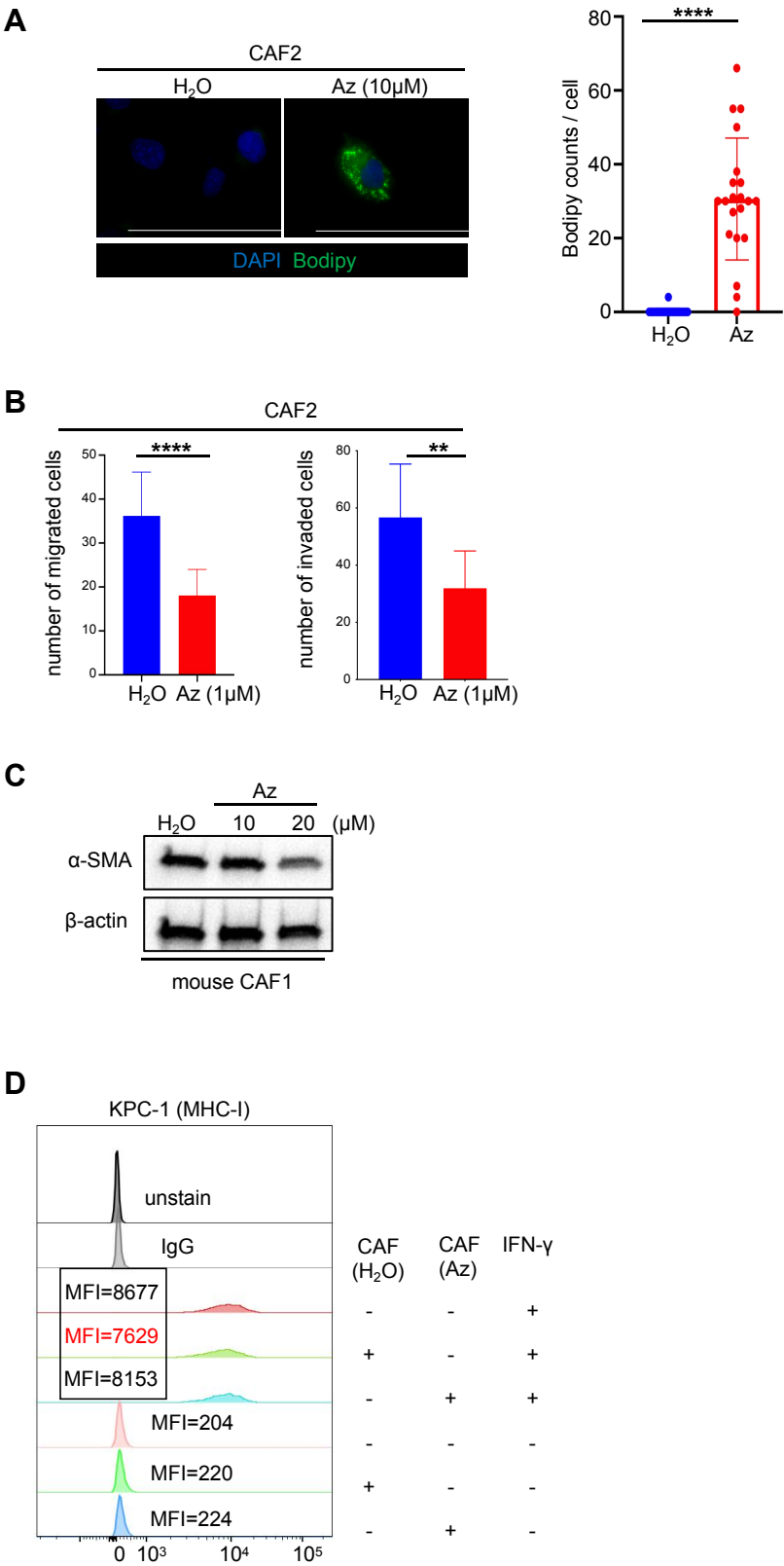

Supplement: Supplementary file 5 — Additional file 5: Supplementary Fig. S5. (A) Representative image of CAF2 stained with BODIPY. Scale bar = 100 µm. (B) The effect of treatment on migration and invasion of CAF2. (C) α-SMA protein expression in whole lysis of mouse CAF1. (D) FCM of MHC-I expression in each group. Error bars, mean ± SD (A,B); **p<0.01, ****p<0.0001. CAF2, human cancer-associated fibroblast 2; α-SMA, α-smooth muscle actin; CAF1, cancer-associated fibroblast 1; MHC-I, major histocompatibility complex class I. [file 13046_2024_3060_MOESM5_ESM.pdf]

Supplementary Figure 6

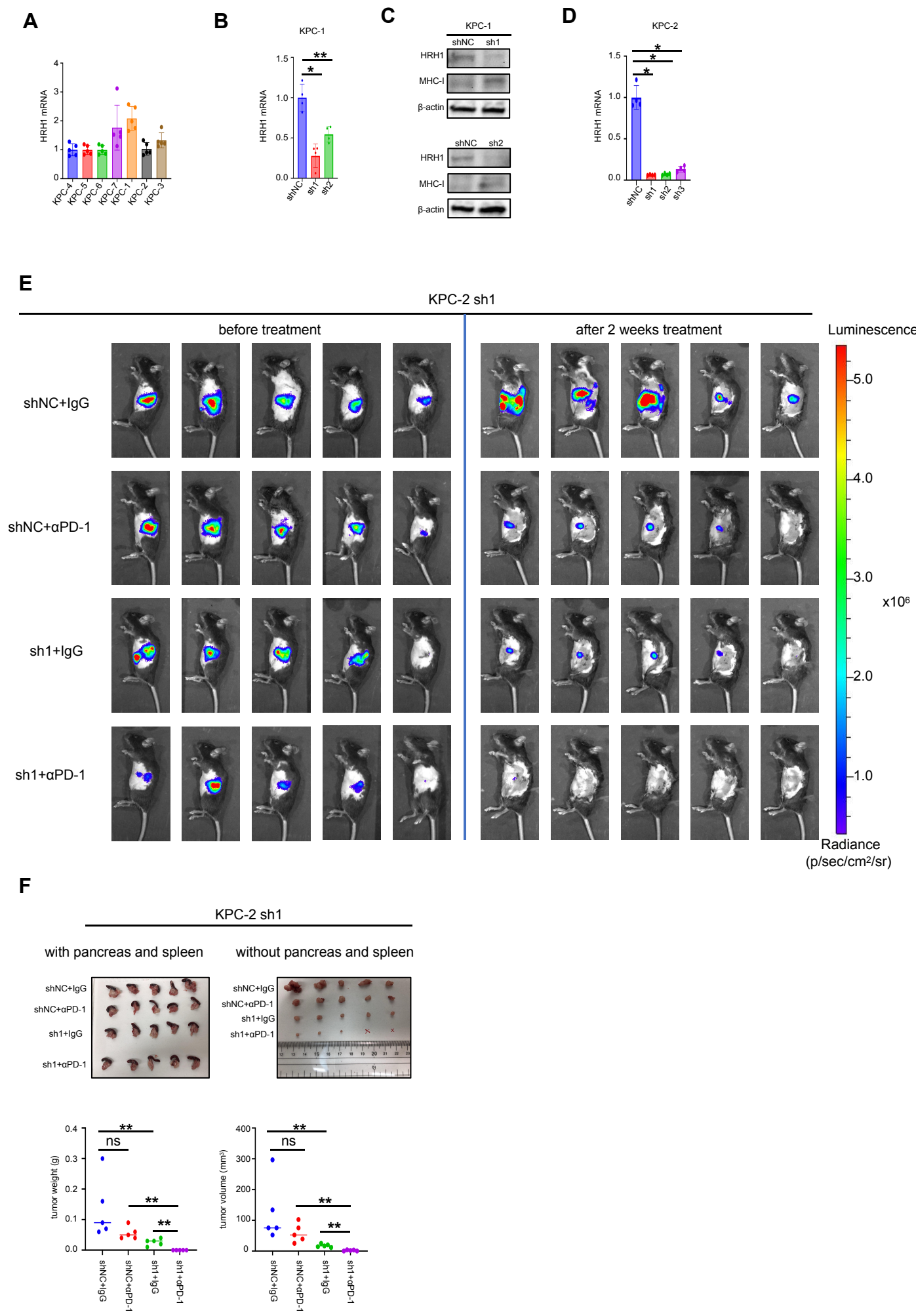

Supplement: Supplementary file 6 — Additional file 6: Supplementary Fig. S6. (A) HRH1 mRNA expression in mouse pancreatic cancer cell lines (KPC-1 to KPC-7), n = 5 per group. (B) HRH1 mRNA expression in KPC-1 shNC and shHRH1 (sh1, sh2), n = 4 per group. (C) HRH1 and MHC-I protein expression in whole lysis of KPC-1 shNC and shHRH1 (sh1, sh2). (D) KPC-2 shNC and shHRH1 (sh1, sh2, sh3), n = 4 per group. (E, F) The orthotopic co-transplanted syngeneic tumors (luciferase-expressing KPC-2 shNC and shHRH1 (sh1)) for treatment of 2 weeks; (E) bioluminescent images of KPC-2 tumors; (F) tumor picture, volume, and weight. Median (F); error bars, mean ± SD (A,B,D); *p<0.05, **p<0.01. HRH1, histamine receptor H1; MHC-I, major histocompatibility complex class I; H&E, hematoxylin and eosin. [file 13046_2024_3060_MOESM6_ESM.pdf]
