# Supplementary material for: Blockade of histamine receptor H1 augments immune checkpoint therapy by enhancing MHC-I expression in pancreatic cancer cells
Source: J Exp Clin Cancer Res. 2024 May 8;43:138. doi: 10.1186/s13046-024-03060-5 (PMC11077718; doi:10.1186/s13046-024-03060-5)
Supplement: Supplementary file 7 — Additional file 7: Supplementary Table S1. Primers used for RT-PCR. [file 13046_2024_3060_MOESM7_ESM.docx]

**Supplementary Table S1. Primers used for RT-PCR.**

| **human primers** | **Forward sequence 5’-3’** | **Reverse sequence 5’-3’** |
| --- | --- | --- |
| HRH1 | GCTGGGCTACATCAACTCCAC | CCCTTAGGAGCGAATATGCAGAA |
| NLRC5 | GTTCTTAGGGTTCCGTCAGCG | CAGTCCTTCAGAGTGGCACAGAG |
| HLA-A | AGATACACCTGCCATGTGCAGC | GATCACAGCTCCAAGGAGAACC |
| HLA-B | CTGCTGTGATGTGTAGGAGGAAG | GCTGTGAGAGACACATCAGAGC |
| HLA-C | GGAGACACAGAAGTACAAGCGC | ACATCCTCTGGAGGGTGTGAGA |
| B2M | CCACTGAAAAAGATGAGTATGCCT | CCAATCCAAATGCGGCATCTTCA |
| CANX | GCTGGTTAGATGATGAGCCTGAG | ACACCACATCCAGGAGCTGACT |
| CALR | TCAAGGAGCAGTTTCTGGACGG | GCATCCTGGCTTGTCTGCAAAC |
| TAPBP | GAGCCTGTTCTCATCACCA TGG | GTAGGCAAAGCTCAAGTCCAGC |
| TAP1 | GCAGTCAACTCCTGGACCACTA | CAAGGTTCCCACTGCTTACAGC |
| TAP2 | ATGCCCTTCACAATAGCAGCGG | CCAAAACTGCGAACGGTCTGCA |
| ERAP1 | GTCTGTCAGTGTGACCCATCCT | CTGAGCAGGATTTTCCACAGGTG |
| SEC61B | CCGCACAACCTCGGCAGGCA | CAGAAGCGATGAACAGAAGACTC |
| SEC61G | GCAGTTTGTTGAGCCAAGTCG | CCAGCCGAATGGAGTCCTT |
| PDIA3 | CCATGCCCTAAGGATGGGTTC | CGTGATGCCACACCCTTGA |
| BCAP31 | AAGCAGTCTGAGGGCCTCAC | TTCTTGTCCATGGGACCATCTAC |
| SREBF2 | TGTGTATGTCCTGTGCCTTTTC | TGGGACACAGTGACTGATTGAT |
| MSMO1 | TCTTTGGGCATGGGTGAC | TGATGCCGAGAACCAGCATA |
| DHCR7 | AATGCCATGGTCCTGGTCAA | CAATGGTCTTCAGGTACCAGGTTTC |
| GAPDH | GCACCGTCAAGGCTGAGAAC | TGGTGAAGACGCCAGTGGA |
|  |  |  |
| **mouse primers** |  |  |
| HRH1 | GTCACGAAGCACATCTGTCCAC | CCATGCCTTCAACAGGATTCA |
| GAPDH | TGTGTCCGTCGTGGATCTGA | TTGCTGTTGAAGTCGCAGGAG |
