# Supplementary material for: Blockade of histamine receptor H1 augments immune checkpoint therapy by enhancing MHC-I expression in pancreatic cancer cells
Source: J Exp Clin Cancer Res. 2024 May 8;43:138. doi: 10.1186/s13046-024-03060-5 (PMC11077718; doi:10.1186/s13046-024-03060-5)
Supplement: Supplementary file 8 — Additional file 8: Supplementary Table S2. Overlapping up-regulated genes of human cell lines. [file 13046_2024_3060_MOESM8_ESM.docx]

**Supplementary Table S2. Overlapping up-regulated genes of human cell lines.**

| **Microarray** | | |
| --- | --- | --- |
| **92 up-regulation genes** | | |
| ACAT2 | HMGCS1 | ORAI3 |
| ACSS2 | HOXB9 | PDE4DIP |
| AKAP12 | HRK | PFKFB2 |
| ALDOC | HS1BP3 | PKD1L2 |
| AMDHD2 | ID2 | PLCG2 |
| ATP6V0E2-AS1 | IDI1 | PLEKHM1 |
| BEX2 | IRS2 | PLEKHO1 |
| BRI3 | ISG20 | PRXL2A |
| C15orf48 | KCP | PSAP |
| C1orf54 | KIF3C | RRAGC |
| CD63 | KLF6 | RRAGD |
| CLCN7 | LGALSL | RTN4R |
| CLIP2 | LOC105376311 | SAT2 |
| CTSA | LOC105378663 | SERPINA3 |
| CXCL2 | LY96 | SH2D5 |
| CYP51A1 | MAFF | SHC2 |
| DHCR24 | MAP1LC3B | SLC16A13 |
| DHCR7 | MCOLN3 | SLC30A3 |
| EBP | MFGE8 | SREBF2 |
| FDFT1 | MOSPD1 | TM4SF19 |
| FDPS | MROH1 | TMEM170B |
| FDPSP2 | MSMO1 | TOLLIP |
| FGFR1 | MVD | TP53INP1 |
| FLCN | MXD1 | TP53INP2 |
| FNIP2 | NAGK | TPM1 |
| GDF15 | NCR3LG1 | TPP1 |
| GNPDA1 | NDRG1 | UAP1L1 |
| GPNMB | NDRG4 | UCA1 |
| HDAC11 | NEURL3 | UPP1 |
| HK2 | NPC2 | VAT1 |
| HKDC1 | NT5DC4 |  |
